# Use of Modified Phenolic Thyme Extracts (*Thymus vulgaris*) with Reduced Polyphenol Oxidase Substrates as Anthocyanin Color and Stability Enhancing Agents

**DOI:** 10.3390/molecules201219854

**Published:** 2015-12-14

**Authors:** Oscar Aguilar, Carmen Hernández-Brenes

**Affiliations:** Centro de Biotecnología-FEMSA, Escuela de Ingeniería y Ciencias, Tecnológico de Monterrey, Campus Monterrey, Ave. Eugenio Garza Sada 2501 Sur, Monterrey, NL 64849, Mexico; alex.aguilar@itesm.mx

**Keywords:** polyphenoloxidase (PPO), thyme extracts, anthocyanins, phenolics, copigmentation

## Abstract

Residual enzymatic activity in certain foods, particularly of polyphenoloxidase (PPO), is responsible for the majority of anthocyanin degradation in food systems, causing also parallel losses of other relevant nutrients. The present work explored the feasibility of modifying phenolic profiles of thyme extracts, by use of chromatographic resins, to obtain phenolic extracts capable of enhancing anthocyanin colour and stability in the presence of PPO activity. Results indicated that pretreatment of thyme extracts with strong-anion exchange resins (SAE) enhanced their copigmentation abilities with strawberry juice anthocyanins. Phenolic chromatographic profiles, by HPLC-PDA, also demonstrated that thyme extracts subjected to SAE treatments had significantly lower concentrations of certain phenolic compounds, but extracts retained their colour enhancing and anthocyanin stabilization capacities though copigmentation. Additional testing also indicated that SAE modified extract had a lower ability (73% decrease) to serve as PPO substrate, when compared to the unmodified extract. Phenolic profile modification process, reported herein, could be potentially used to manufacture modified anthocyanin-copigmentation food and cosmetic additives for colour-stabilizing applications with lower secondary degradation reactions in matrixes that contain PPO activity.

## 1. Introduction

One of the main disadvantages for the general use of anthocyanins as food colorants is their low stability. In fact, the color stability of anthocyanins depends on a combination of several factors, which include their main structures, concentration, pH, temperature, the presence of complexing agents (phenols, metal ions), oxygen, light, enzymes, and interaction with other food components, among others [1,2,3,4]. It has been proved by several authors that anthocyanins have radical scavenging capacity, which confers them antioxidant properties [5,6,7].

Processing of fresh fruits is always accompanied by a consequent cellular stress and breakdown by tissue damage. Therefore the activity of many enzymes increases as a result of increased permeability and mixing of enzymes and substrates that, in intact tissues, are entrapped into vacuoles and other cellular compartments [8]. Polyphenol oxidase (PPO) acts on anthocyanins in the presence of *o*-diphenols, via a coupled oxidation mechanism [2], and can catalyze various enzymatic browning reactions affecting the quality and acceptability of processed products. In many fruits, including strawberry, PPO activity is responsible for red color losses due to degradation of anthocyanins [9].

It is well known nowadays that the colorless polyphenols (flavones, flavonols, tannins esters of cinnamic and benzoic acids,) are capable of forming complex non-covalent molecular adducts with the flavylium ion nuclei of anthocyanins, which is flat and rich in π electrons; an interaction that increases color intensity due to the formation of colorful aggregates [10,11]. These copigments include a variety of structurally related and unrelated compounds, such as flavonoids, amino acids, non-flavonoid phenolics and organic acids [12].

A wide range of phenolic extracts of vegetable origin contain flavonoids as their main constituents in the forms of aglycones, such as quercetin, myricetin, kaempferol and their glycosides [13]. Some species such as thyme (*Thymus vulgaris*) and rosemary (*Rosmarinus officinalis*) are widely known to have high antioxidant capacity, and some methylated flavones with potent antioxidant activity have been isolated from thyme. Although some phenolic compounds are not directly involved in the enzymatic browning, they can act as polyphenol oxidase synergists. Shannon and Pratt reported that *p*-coumaric acid is an uncompetitive inhibitor of apple PPO, and that it catalyzes the degradation of chlorogenic acid [14,15].

The formation of complexes between anthocyanins and copigments, under acidic conditions, improves the visual quality and color stability of foods that contain them. The stability of copigmented anthocyanins in the presence of ascorbic acid is known to have a chemoprotective effect during food processing [16,17]. Several substances such as quercetin-5-sulfonate, chlorogenic acid, morin, rutin, quercetin, *etc.*, have been identified as good copigments [18]. However, several of these compounds, mainly phenolic acids such as caffeic, chlorogenic and protocatechuic acids, also act as polyphenol oxidase substrates [19].

Several strategies have been devised to avoid the accumulation of specific phenolic compounds. Some involve the use of 2-aminoindane-2-phosphonic acid to inhibit phenylpropanoid biosynthesis in some tissues, however, even when this results effective in decreasing enzymatic browning, it also decreases the antioxidant properties of the plants [20]. In other studies, Guillen *et al.*, reported a fractionation process for wine phenolics using two solid-phase extraction cartridges; the first one was a C18 resin, followed by an anion exchange resin [21]. Although organic solvents can be used for elution of adsorbed polyphenols from C18 cartridges, the selectivity of such solvents is not enough to achieve molecular separation based only on minor structural differences in polarity. The use of ionic resins for fractionation of phenolic extracts in food applications has been previously reported as an analytical sample preparation stage and for targeted fractionation [21,22,23]. Similarly, a study by Petelnic *et al.* showed that fractionation of phenolic extracts has an effect on the antioxidant capacity when testing the uptake of specific compounds in yeast cells [24].

The use of natural phenolic additives in food processing has been suggested as an alternative to chemical additives due to their properties as antioxidants and copigments for certain food matrices [5,16,17]. In the present work, the use of treatment steps for extracts with anion exchange chromatography were explored in an attempt to fractionate thyme phenolics, without affecting their ability to stabilize anthocyanins through copigmentation. Potential uses of extracts modified by anion exchange chromatography as anthocyanins copigments, were also validated at equivalent phenolic levels. Phenolic profile analyses of modified extracts and their ability to serve as enzymatic substrates were explored as well in the present work, to verify that the desired functional modifications of the extracts were attained.

## 2. Results and Discussion

### 2.1. Modified Extracts by Anionic Exchange Chromatography Pre-Treatment

Thyme phenolic extracts were obtained by a common commercial procedure described by Bailey *et al.* [25]. The process yields UNP, a watery extract with high concentrations of phenolic compounds (Table 1) that has been recognized as a source of anthocyanin-stabilizing compounds with antioxidant properties. However, the use of UNP thyme extracts as a food additive has been limited by potential increases in its enzymatic-browning reactions [26,27,28]. Strategies suggesting the use of solid phase extraction for the modification of phenolic profiles have been reported in the literature for several decades and reflect an ongoing interest in developing simple steps to improve and expand the uses of natural extracts enriched in phenolics. As a primary result from the present work, the use of anionic resins for the fractionation of thyme extracts had a direct impact on the concentration of total phenolics (measured as gallic acid equivalents, GAE), reducing their concentration by 24.3% when the extract was treated with a weak anionic exchanger (WAE), and by 13.8% for the extract treated with a strong anionic exchanger (SAE), as indicated in Table 1. These differences in phenolic contents can be attributable to the different functional groups present in each ion exchange resin and their differential affinity for the charged species present in the thyme extracts, particularly phenolic acids. Further HPLC-PDA analysis also indicated that phenolic profiles of the extracts were modified, as will be described in later sections.

**Table 1:** Effect of anionic resin treatments on the phenolic contents of water-soluble thyme extracts.

Extract ^1^	Total Soluble Phenolics (mol/Kg) ^2^
**UNP**	0.812 ± 0.006 ^3^
**WAE**	0.614 ± 0.007
**SAE**	0.700 ± 0.001

^1^ UNP = not processed through anion exchanger, WAE = processed through weak anion exchanger, SAE = processed through strong anion exchanger; ^2^ Expressed as moles of gallic acid equivalents per Kg of thyme; ^3^ Average of three repeated purifications.

### 2.2. PPO Activity in the Presence of Modified Thyme Extracts

Jacobo-Velazquez *et al.* have reported the use of rosemary and thyme phenolic extracts in an avocado matrix in an attempt to decrease residual oxidative enzyme activities in high hydrostatic pressure processed foods. However, it was reported that the addition of such natural extracts contributed to enzymatic browning by providing potential substrates for polyphenol oxidase, an enzyme that regains its activity days after processing during the shelf-life of the product [29,30].

**Figure 1:**
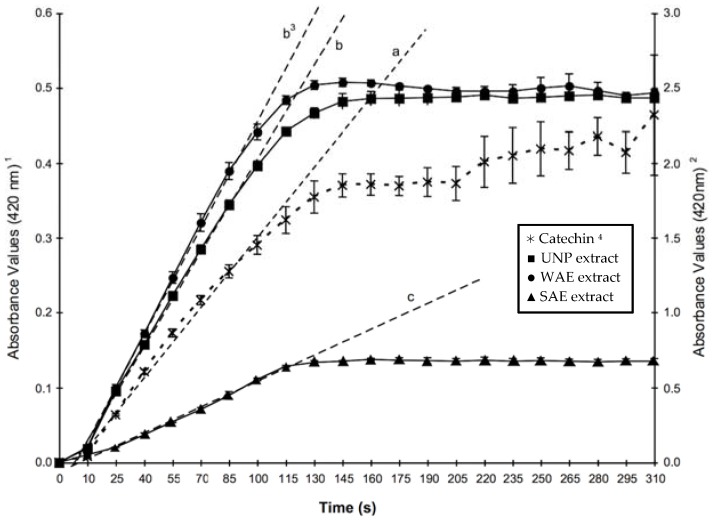
Enzyme activity of polyphenoloxidase (PPO, 300 U/mL) in model assays (30 °C, pH 6.5) formulated with different thyme extracts as enzyme substrates and catechin shown as reference. (--) Trend lines. Bars represent standard error of the mean (*n* = 3). **^1^** Absorbance values (left axis) measured in model systems that contained thyme extracts as PPO substrates. ^2^ Absorbance values (right axis) measured in model systems that contained catequin (5 mM) as PPO substrate. ^3^ Trend lines with different letters indicate that treatment reactions rates calculated by the initial velocity method are significantly different (LSD test, *p* < 0.05). ^4^ (✲) Catechin; (■) UNP extract, 27.1 mM total soluble phenolics (TSP); (●) WAE extract, 20.5 mM TSP; (▲) SAE extract, 23.3 mM TSP as gallic acid equivalents.

In the present study, modified thyme extracts were evaluated as potential PPO substrates, to determine if the chromatographic pretreatments had any effect on the removal of specific substrates, previously reported to be present in the extracts. Data on initial formation rates of PPO products (in this case the *o*-quinones) are shown in Figure 1, for the three different extracts evaluated in the study (UNP, WAE and SAE). No significant differences were observed between the enzymatic rates of UNP and WAE treatments, whereas SAE extracts showed a 73% decrease in PPO activity. Higher product formation rates for WAE and UNP, shown in Table 2, also suggested that the enzyme had greater amounts of substrates or necessary cofactors, in those extracts, to perform its activity.

Data also indicated that the strong anionic resin treatment, which produced SAE, was able to partially remove compounds identified as PPO substrates, such as catechin, epicatechin, caffeic, *p*-coumaric and gallic acids, among others [19]. Another possible explanation for this behavior would be the potential removal of enzyme activators that may have been retained by the strong anionic resin.

**Table 2:** Initial rates for quinone formation by polyphenoloxidase (PPO) in model systems using thyme extracts and catechin as enzyme substrates.

PPO Substrate	Total Soluble Phenolics (mM) ^1^	Initial Formation Rate (μmol/min) ^2,4^
***UNP***	95.4	0.151 ± 0.003 b ^3^
***WAE***	57.4	0.171 ± 0.004 b
***SAE***	61.4	0.041 ± 0.002 c
***Catechin (CAT)***	44.4	0.568 ± 0.025 a

^1^ Expressed as gallic acid equivalents; ^2^ Obtained from the slope of absorbance *vs.* time plot; ^3^ Values with different letters are significantly different (LSD test, *p* < 0.05) and indicate the effect of different resins; ^4^ Standard error for rates obtained as the average of three measurements.

### 2.3. Copigmentation Using Modified Thyme Extracts

**Figure 2:**
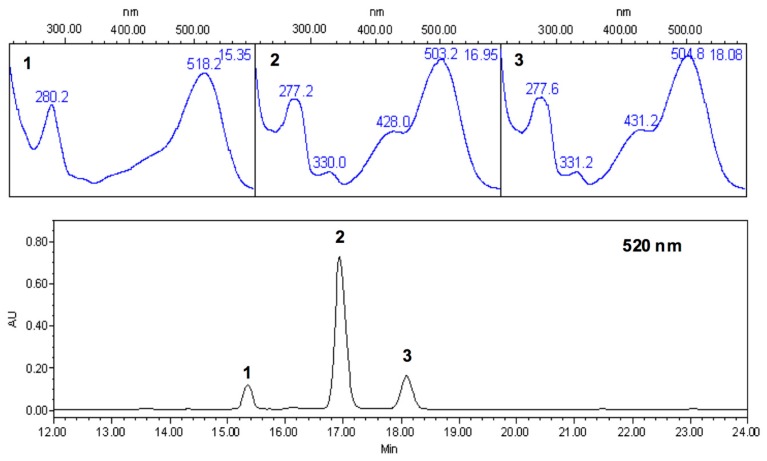
HPLC-PDA chromatogram of anthocyanins present in strawberry juice (*Fragaria anannassa* L.) @ 520 nm, and UV-Vis spectra showing their chemical identity: (**1**) cyanidin-3-glucoside; (**2**) pelargonidin-3-glucoside; (**3**) possible pelargonidin-3-rutinoside.

Quantification of anthocyanins in strawberry juice, using a pH differential method, showed a concentration of 16 ± 0.12 mg/100 g fruit, expressed as pelargonidin-3-glucoside. PDA-HPLC chromatographs of strawberry juice anthocyanins confirmed the identity of the three glycoforms present, as reported by Garcia-Viguera and Bridle [31]. Thus, the anthocyanins were primarily composed by pelargonidin-3-glucoside (72.3%), small amounts of cyanidin-3-glucoside (10.4%), and pelargonidin-3-rutinoside (17.3%) (Figure 2).

Strawberry juice copigmentation systems were formulated in different molar ratios (0 to 100) as indicated in Table 3, considering total anthocyanin and total phenolic contents of each extract, as described in the Experimental Section. Several studies have demonstrated the effects of varying the copigment/pigment molar ratios on the spectral properties of malvidin 3,5-diglucoside copigmented with caffeic and ferulic acids, and have also characterized the impacts by measuring hyperchromic and bathochromic shifts in their systems [32,33]. The present work also evaluated those parameters in strawberry juice copigmented with thyme extracts, with the results shown in Table 3. Bathochromic changes of copigmented strawberry juice indicated that a maximum shift occurred at a ratio of 75, after which no significant difference was observed. Many authors have also reported this type of apparent saturation behavior [10,17,32,34,35]. The hyperchromic shifts of the strawberry juice showed a different behavior, regardless the molar ratio in which the shift was evaluated; SAE treatments exhibited a significantly higher increase in absorbance than the other treatments, results that were highly desirable since they indicate better copigmentation capacities for SAR-modified extracts and thus, potentially greater stabilizing properties.

**Table 3:** Effect of modified thyme extracts on the spectroscopic characteristics of strawberry (*Fragaria ananassa*) juice systems.

	Thyme Extract	Molar Ratio Copigment/Pigment									
		0		25		50		75		100	
**Batochromic shift (nm) ^2^**	**UNP**	0.0	C ^1^	8.0	B a ^1^	13.0	A a	13.3	A b	14.0	A b
	**WAE**	0.0	D	8.0	C a	11.3	B a	13.7	A ab	12.7	AB b
	**SAE**	0.0	D	9.3	C a	12.0	B a	15.3	A a	16.7	A a
**Hiperchromic shift (%) ^3^**	**UNP**	0.0	C	47.0	A ab	50.2	A b	30.4	B c	33.6	B b
	**WAE**	0.0	C	32.5	B b	48.8	AB b	53.7	A b	40.2	AB b
	**SAE**	0.0	E	56.8	D a	73.0	C a	98.5	B a	116.8	A a
Lightness (L*)	**UNP**	39.1	A	30.5	B a	28.1	C a	26.1	D a	25.1	E a
	**WAE**	37.5	A	28.7	B b	25.9	C b	22.0	D c	21.7	D c
	**SAE**	38.6	A	28.7	B b	25.9	C b	24.2	D b	23.6	E b
Chroma Value (C*)	**UNP**	48.9	A	39.8	B a	34.8	C a	26.2	D a	22.0	E a
	**WAE**	48.8	A	36.2	B b	28.8	C c	20.0	D b	19.4	D b
	**SAE**	50.4	A	40.5	B a	33.1	C b	26.4	D a	22.2	E a
Hue Angle (h*)	**UNP**	41.2	A	25.7	B b	22.7	C b	20.8	D b	19.7	E c
	**WAE**	41.6	A	24.6	B c	21.5	C c	20.8	D b	20.3	D b
	**SAE**	43.0	A	27.3	B a	24.2	C a	21.8	D a	21.2	E a

^1^ Values with different lowercase letters within a column for each response variable are significantly different (LSD test, *p* < 0.05). Values with different uppercase letters between columns for each treatment and response variable are significantly different (LSD test, *p* < 0.05); ^2^ Relative to the λ_max_ of strawberry juice at level 0 = 498 nm; ^3^ Calculated as the percentage increment in absorbance of copigmented juice (at λ_max_) relative to juice without thyme extract.

Both anion resin treatments decreased the total phenolic contents of the extracts, which resulted in an increase in thyme extract volumes required to achieve the desired molar pigment:copigment ratios. However, the use of additional copigment (thyme extract) volumes did not cause an increase in absorbance for WAE treatments, whereas for SAE treatments increases in absorbance by up to a 116% were observed. Results that suggested that spectral changes were not proportional to volume increases, particularly not for WAE extracts that contained the lowest phenolic contents (Table 1).

Changes in color “intensity” or hyperchromism corresponded to variations arising from the modification of two instrumental color attributes that included *lightness* (L*) and *chroma* (C*), due to the slightly brown coloration of the thyme extracts. Average *hue* (h*) value for the treatments shifted from 42, (orange in the color space) for the system without copigment, to up to 20.4 (red colors) in the CIELAB system. In this series of treatments the behavior observed demonstrated that the greater the amount of copigment added, the higher were the overall effects on the instrumental color [35,36].

### 2.4. Anthocyanins Total Monomer, Polymer Color and Color Density

Comparisons of the abilities of the three extracts to modify color intensity and polymerization behavior of anthocyanins are shown in Table 4. Decreases in concentrations of total monomeric anthocyanins at a ratio of 100 were observed, when compared to treatments without copigment (level 0); reductions of 28.6% for UNP extract, 16.2% for WAE extract and 13.6% for SAE extract were observed. The formation of compounds by association of anthocyanins with other molecules present in the extracts could be the reason for the decrease in the amount of monomeric anthocyanins in the presence of extracts, due to the formation of covalent bonds anthocyanin-copigment [37,38,39], preventing copigmentation and decreasing the total monomeric anthocyanins in the juice.

**Table 4:** Effect of thyme phenolic extracts on the physicochemical properties of strawberry juice systems (*Fragaria anannassa* L.).

	Thyme Extract	Molar Ratio Copigment/Pigment									
		0		25		50		75		100	
**Monomeric Anthocyanins ^2^ (mg/L)**	**UNP**	85.28	A ^1^	78.72	B b ^1^	73.67	C b	62.27	D b	60.89	D b
	**WAE**	94.47	A	92.11	A a	83.71	B a	80.11	BC a	79.12	C a
	**SAE**	94.76	A	82.04	B ab	80.77	B a	79.56	B a	81.86	B a
**Color Density**	**UNP**	1.91	E	3.10	D c	4.36	C b	5.63	B a	7.12	A a
	**WAE**	2.14	D	3.49	C a	5.13	B a	5.79	B a	6.83	A a
	**SAE**	1.79	E	3.22	D b	4.41	C b	5.84	B a	7.15	A a
**Polymeric Color (%)**	**UNP**	18.64	E	32.81	D a	38.34	C a	49.18	B a	55.51	A a
	**WAE**	17.08	D	30.88	C a	41.62	B a	42.71	AB b	48.37	A b
	**SAE**	26.40	C	31.90	C a	39.72	B a	45.90	A ab	45.77	A b

^1^ Values with different lowercase letters within a column for each response variable are significantly different (LSD test, *p* < 0.05). Values with different uppercase letters between columns for each treatment and response variable are significantly different (LSD test, *p* < 0.05). ^2^ As pelargonidin-3-glucoside equivalents.

Color density exhibited an opposite behavior than percent of monomeric anthocyanins; since addition of copigmentation treatments increased concentrations of phenolic compounds in the systems, which overlapped anthocyanin maximum absorbance values with those of compounds present in thyme extracts. Similarly, gradual increases in concentrations of phenolic compounds, contained in thyme extracts, also increased percent polymeric color values (Table 4); possibly due to a protective effect of the π overlap of anthocyanins with the copigments, making them resistant to SO_2_ bleaching.

### 2.5. Characterization of Thyme Extracts by HPLC-PDA

**Figure 3:**
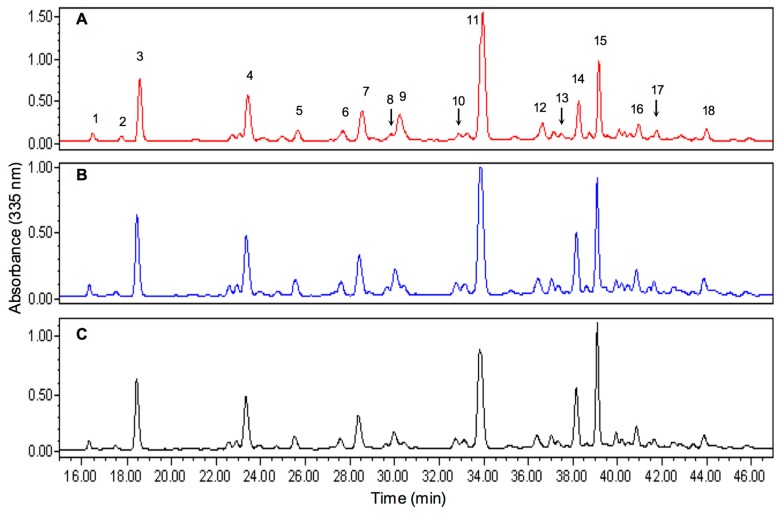
HPLC-PDA chromatograms (335 nm) of flavonoids present in thyme (*Thymus vulgaris* L.). (**A**) UNP extract; (**B**) WAE extract; (**C**) SAE extract. Peak assignments: 1. kaempferol-3-glucoside or probable 3,7-*O*-glycoside; 2. coelution; 3. apigenin-8-C-glucoside; 4. naringenin derivative; 5. glycosilated apigenin (ring B); 6. apigenin; 7. luteolin derivative (ring A); 8. possible flavone; 9. hesperetin; 10. probable diglycoside of peak 17; 11. rosmarinic acid derivative; 12. unknown, probable coelution; 13. naringenin; 14. luteolin; 15. rosmarinic acid; 16. glycoside of peak 17; 17. possible flavone; 18. possible flavone.

Determining the phenolic composition of thyme extracts by HPLC-PDA was insightful to understand the differences in their copigmentation behavior and ability to serve as PPO substrates. Phenolic profiles were quantified (ppm) in equivalents of three commercial standards at three different selected wavelengths, which included 280 nm for gallic acid, 335 nm for luteolin and 380 nm for quercetin [40]. A comparison between the flavonoid chromatograms for the three extracts at λ = 335 nm is shown in Figure 3, at this wavelength most of the peaks showed at least a maximum absorbance. It can be observed that the difference in the profiles of the three chromatograms is minimal, except for the height of the peaks, and therefore its area.

The chemical identity of each peak (Table 5) showed that all extracts contained several glycosylated forms of the same compound as in the case of apigenin and luteolin with different retention times [41].

**Table 5:** Chemical identification of peaks obtained by HPLC-PDA in thyme extracts.

Peak (Retention Time, min) ^1^	λ_max_ (nm) Absorption Bands ^2^	Chemical Identity	Identification Method ^3^
1 (16.5)	268.7, 349.5	Kaempferol-3-glucoside or probable 3,7-*O*-glycoside	A, B
2 (17.72)	297.1, 325.1	Coelution	A
3 (18.57)	216.8, 273.4, 335.2,	Apigenin-8-C-glucoside	A, B
4 (23.44)	216.8, 282.9, 330.4s	Naringenin derivative	A, B
5 (25,64)	268.7, 335.2	Glycosilated apigenin (ring B)	A, B
6 (27.67)	268.7, 340.0	Apigenin	A, B
7 (28.54)	268.7, 349.5	Luteolin derivative (ring A)	A, B
8 (29.83)	282.9, 335.2	Possible Flavone	
9 (30.44)	282.9, 335.2	Hesperetin	A, C
10 (32.9)	282.9, 340.0	Probable di-glycoside of peak 17	
11 (33.93)	330.4	Rosmarinic acid derivative	A, B
12 (36.62)	287.6, 325.7	Unknown, probable coelution	A, B, C
13 (37.48)	287.6	Naringenin	
14 (38.26)	254.5, 349.5	Luteolin	A, B, C
15 (39.17)	330.4	Rosmarinic acid	A, B, C
16 (40.96)	282.9, 340.0	Glycoside of peak 17	
17 (41.52)	282.9, 340.0	Possible Flavone	
18 (43.97)	287.6, 344.7	Possible Flavone	

^1^ Assigned according to chromatogram (Figure 3); ^2^ Taken from absorption spectra of eack peak, s = shoulder; ^3^ Identification by direct comparison to authentic standards (**A**); by spectral interpretation (**B**) and, by retention time (**C**).

Comparative analysis by overlaying the chromatograms (Figure 3) showed that in terms of the elution profile of the compounds, there were minimal differences between treatments, almost unnoticeable; however, quantification results indicated significant differences for particular compounds. As previously mentioned, in reference to UNP treatments, concentrations of most individual compounds declined in the anion exchange treated extracts (Table 6), observations that were consistent with lower total phenolics, for the same extracts, as determined by Folin-Ciocalteau assay (Table 1).

Quantification of phenolic species identified in the chromatograms showed few significant differences, except for certain specific compounds that are shown in Table 6. Hesperetin concentrations decreased significantly with the WAE treatments, when compared to UNP. This trend was also observed for almost all peaks shown in Table 6, indicating that apparently WAE resin reduced levels of specific compounds. For rosmarinic acid and luteolin, which are good copigments, something similar happened, as their concentrations in WAE-treated extracts were lower than in those treated with SEA resins. It is noteworthy that glycosylated derivatives of both compounds showed no differences between both resins, probably due to steric hindrance, charge distribution, or even polarity in the selectivity of the anion resin.

An important feature observed by comparing concentrations between the anion exchange treatments, is that some of the compounds typically identified as good copigments (luteolin and rosmarinic acid) were unaffected or minimally affected by the treatment with the SAE resin. This allowed SAE-modified extracts, to retain their ability to act as copigments, as confirmed in copigmentation assays (Table 3 and Table 4) where, in fact an improvement was observed for certain parameters by using SAE-modified extracts. The removal of specific compounds by the resins without affecting other components in the extracts, affected competition between copigment molecules for anthocyanins, giving them more opportunity to form π-π stacks with anthocyanins and thus produce greater bathochromic and hyperchromic shifts. These observations appeared to be supported by the Pearson correlation coefficients, but cannot be conclusively proven [42]. For example, concentrations of peak 13, identified as naringenin, were diminished by the SAE resin treatment (Table 6), which in turn could be contributing to the increased copigmentation capacities of the extracts (r = −0.92 for hyperchromic shift at level 100). Such a decrease in naringenin concentration was also related to the decrease in the PPO enzymatic activity of the extract SAE (r = 0.89). Similarly, peak 10, which was not fully identified, but was tentatively thought to be a flavone glycoside, presented a similar behavior; when its concentration decreased, the copigmentation capacity was improved (r = −0.83), and the PPO activity of the extract was also reduced (r = 0.72).

**Table 6:** Comparative analysis of phenolic composition in thyme extracts by HPLC-PDA.

Peak (Retention Time, min)	Chemical Identity	Concentration in ppm ^1^		
		UNP	WAE	SAE
5 (25,64)	Glycosylated apigenin (ring B)	126.2 a ^2^	99.0 b	101.7 b
6 (27.67)	Apigenin	157.8 a	114.9 b	94.4 b
7 (28.54)	Luteolin derivative (ring A)	329.8 a	267.5 b	274.5 b
9 (30.44)	Hesperetin	512.9 a	427.9 b	441.4 a,b
10 (32.9)	Probable diglycoside of peak 17	118.9 a	67.8 b	89.0 b
11 (33.93)	Rosmarinic acid derivative	764.1 a	480.2 b	481.0 b
13 (37.48)	Naringenin	319.8 a	217.7 c	254.2 b
14 (38.26)	Luteolin	341.2 a,b	318.2 b	360.1 a
15 (39.17)	Rosmarinic acid	422.0 a	365.5 b	442.5 a

^1^ Expressed as gallic acid equivalents, luteolin equivalents or quercetin equivalents, according to its chemical nature; ^2^ Values with different letters for the same compound are significantly different (LSD test, *p* < 0.05).

The implementation of the modified commercial process would imply the introduction of an additional adsorption process that could be coupled to or even substituting C-18 adsorption step in order to inexpensively obtain commercial quantities of this GRAS food additive. Further analysis should consider the introduction of this fractionation method at either the initial stages of the process, or as in this case, after the removal of methanolic fractions. In general, SAE treatments resulted in better copigments for strawberry anthocyanins due to a combination of factors. They contained significantly lower concentrations of phenolic compounds that served as PPO substrates, but extracts retained phenolic compounds that served as good copigments; therefore SAE treatment retained and even improved the extract’s colour enhancement and anthocyanin stabilization capacities. Such low enzyme activity without compromising stabilization ability is a desirable characteristic to overcome the potential cost of introducing a chromatographic resin in its production process.

## 3. Experimental Section

### 3.1. Materials

HPLC calibration standards, Folin-Ciocalteau reagent and polyphenol oxidase (E.C. 1.14.18.1) were obtained from Sigma-Aldrich Co. (St. Louis, MO, USA) and Fluka Chemie GmbH (Buchs, Switzerland). Pectic enzymes mixture was purchased from Novozymes (Krogshoejvej, Denmark). The acids and buffers used were obtained from Products Químicos Monterrey S.A. de C.V. (Monterrey, N.L., Mexico), Merck Co. (Darmstadt, Germany) and Desarrollo de Especialidades Químicas, S.A. de C.V. (San Nicolás de los Garza, N.L., Mexico). HPLC-grade solvents were purchased from Fisher Scientific Int. (Winnipeg, MB, Canada). The Sep-Pak C-18 cartridges were from Waters Co. (Milford, MA, USA). The two anion exchange resins Diaion^®^ WA30 and Diaion^®^ PA308 were obtained from Supelco (Bellefonte, PA, USA).

### 3.2. Preparation of Water-Soluble Extracts from Thyme

The preparation of water-soluble thyme extract (unprocessed, UNP) was carried out according to the procedure described by Bailey [25]. Briefly, an infusion of dried thyme leaves (60 g/L) (obtained from a local supermarket) was acidified after boiling and filtered to remove solids. Supernatant was subjected to solid phase extraction to concentrate phenolic compounds using previously activated Sep-Pak C18 cartridges. Phenolic compounds were eluted with acidified methanol (0.01% *v*/*v* HCl). Methanol was removed using evaporation under reduced pressure at 45 °C and −25 mm Hg. A stock solution with 5 g of thyme extract in 50 mL of phosphate buffer pH 6.5, I = 0.05 (identified as extract UNP) was prepared and passed through polypropylene columns loaded with an anion exchanger (BioRad, Hercules, CA, USA). The anion resins used were Diaion^®^ WA30 (WAE) with alkylamine type RxNH_3_OH functional groups and the Diaion^®^ resin PA308 (SAE) with benzyltrimethylamine type RxNR_3_OH functional groups. A flow rate of 4 mL/min was used with 4.6 mL of extract per g. resin. The concentration of total phenolics in thyme extract was determined by the Folin-Ciocalteu method reported by Vinson *et al.* and expressed as gallic acid equivalents (EAG) [43].

### 3.3. Copigmentation Assays

Enzymatically clarified strawberry juice was used for copigmentation assays according to the procedure previously reported by Del Pozo-Insfran, using copigment:pigment molar ratios of 0, 25, 50, 75 and 100 for the two types of processed (WAE, SAE) and unprocessed (UNP) phenolic extracts [16]. These relationships were established based on total concentration of monomeric anthocyanins in strawberry juice measured by a differential pH method [44]. Copigmentation systems were formulated with 3 mL of strawberry juice and copigment volume such as to provide the aforementioned molar ratio according to the concentration of total phenolics in the extract. Systems were completed to 10 mL with 0.02 M pH 3.5 citrate buffer.

Physicochemical changes occurring following the addition of copigments were evaluated as a function of the phenolic extract added to the system and its concentration. The spectral changes were monitored by the increase in absorbance (% hyperchromic shift) and the displacement of the wavelength (λ) of maximum absorbance (bathochromic shift) ranging from 420 to 700 nm [10]. Instrumental CIE color features including lightness (L*), chroma (C*) and hue angle (h*) were measured using a Minolta Chroma Meter CR-300 Series (Minolta Co. Ltd., Osaka, Japan) with a D65 light source and a 10° standard observer. Additionally, the percentage of monomeric/polymeric anthocyanins was determined based on color retention in the presence of sodium metabisulfite, while color density was determined by spectral measurements at 420, 520, and 700 nm according to the method reported by Rodriguez-Saôna *et al.* for all copigment:pigment relations [45].

### 3.4. Polyphenol Oxidase Assay

The spectrophotometric quantification of polyphenol oxidase was made modifying the methods described by Stauffer [19] and Worthington [46]. Briefly, 0.1 mL of copigment were mixed with 2.8 mL of phosphate buffer, pH 6.5, I = 0.05 in a temperature-controlled cell at 30 °C. After temperature equilibration, an aliquot of 0.1 mL of polyphenol oxidase with 300 U/mL was added and the absorbance was monitored at 420 nm in a Model DU 650 spectrophotometer (Beckman^®^, Fullerton, CA, USA). The initial reaction rates were determined considering the molar absorptivity of quinones in phosphate buffer as 5 × 10^3^ M^−1^·cm^−1^ [47].

### 3.5. Chromatographic Profile of Thyme Phenolic Extracts

The determination of the phenolic profile of thyme extracts was performed by RP-HPLC-PDA using the conditions reported by Del Pozo-Insfran *et al.* Two mobile phases consisting of water (phase A) and 60% methanol (phase B) adjusted to pH 2.4 with orthophosphoric acid were used. The separation was performed on a Symmetry C18 column 4.6 mm × 250 mm equipped with a Novapak C18 5 mm guard column (Waters Co.). Samples were diluted 1:1 with 1.2 M HCl solution in methanol HPLC grade and filtered through 0.45 μm PTFE membranes (Gelman, Ann Arbor, MI, USA). The elution gradient consisted of phase B from 0%–30% in 3 min, 30%–50% in 5 min, 50%–70% in 17 min, 70%–80% in 5 min, 80%–100% in 5 min and maintained for 10 min, with a constant flow of 0.8 mL/min [35]. The identification of each peak was performed by comparison with authentic standards (Method A), spectral interpretation (Method B) and retention times (Method C). The chromatograms obtained were analyzed in three wavelengths selected according to the chemical nature: 280 nm for phenolic acids, 335 nm for flavones and 380 for flavonols.

### 3.6. Statistical Analysis

The copigmentation study was designed as a full 3 × 5 factorial including three different extracts (UNP, WAE and SAE) and five molar relations copigment:pigment (0, 25, 50, 75 y 100). Data represent the mean of three replicates. Regression analyses, Pearson correlation coefficients, and analysis of variance (ANOVA) were conducted using JMP software Version 5.0.1.2 (SAS Institute, Cary, NC, USA), with mean separation performed by the least significant difference (LSD) test (*p* < 0.05).

## 4. Conclusions

Pretreatment of thyme extracts with two different chromatographic resins (SAE and WAE) resulted in modified extracts with different phenolic profiles that also displayed a differential ability to provide PPO substrates when compared to the initial thyme extracts. A significant increase in the copigmentation capacities of SAE extracts was observed, results that were mainly attributed to selectivity of the strong resin to remove some specific compounds, whose copigmentation capacity was lower. Increases up to 117% were achieved in the hyperchromic shifts of strawberry anthocyanins, for SAE extracts, compared to increases of 34% achieved with the unmodified extract. The analysis through HPLC-PDA allowed us to determine specific changes in the phenolic profile of the modified extracts, particularly the ability of SAE resins to remove other phenolics and retain copigments (including luteolin and rosmarinic acid), while also decreasing the concentration of specific compounds that are substrates of PPO. Further analysis of the ion exchange process proposed here would involve analyzing the fraction of phenolic compounds retained in the resin, in order to design a better separation strategy to obtain extracts with different phenolic profiles for various applications, without the evident disadvantage of providing substrates for enzymatic oxidation processes.
